# Altered reward system reactivity for personalized circumscribed interests in autism

**DOI:** 10.1186/s13229-018-0195-7

**Published:** 2018-01-30

**Authors:** Gregor Kohls, Ligia Antezana, Maya G. Mosner, Robert T. Schultz, Benjamin E. Yerys

**Affiliations:** 1 Department of Child and Adolescent Psychiatry, Psychosomatics and Psychotherapy, RWTH Aachen University, Aachen, Germany; 2 Department of Psychology, Virginia Polytechnic Institute and State University, Blacksburg, VA USA; 3 Department of Psychology, University of North Carolina at Chapel Hill, Chapel Hill, NC USA; 4 Center for Autism Research, The Children’s Hospital of Philadelphia, 3535 Market Street, Ste 860, Philadelphia, PA 19104 USA; 5 Pediatrics Department, Perelman School of Medicine, University of Pennsylvania, Philadelphia, PA USA; 6 Psychiatry Department, Perelman School of Medicine, University of Pennsylvania, Philadelphia, PA USA

**Keywords:** Autism spectrum disorders, Reward, Motivation, Circumscribed interests, Restricted and repetitive behaviors and interests, Functional magnetic resonance imaging, Striatum, Caudate nucleus, Reward system

## Abstract

**Background:**

Neurobiological research in autism spectrum disorders (ASD) has paid little attention on brain mechanisms that cause and maintain restricted and repetitive behaviors and interests (RRBIs). Evidence indicates an imbalance in the brain’s reward system responsiveness to social and non-social stimuli may contribute to both social deficits and RRBIs. Thus, this study’s central aim was to compare brain responsiveness to individual RRBI (i.e., circumscribed interests), with social rewards (i.e., social approval), in youth with ASD relative to typically developing controls (TDCs).

**Methods:**

We conducted a 3T functional magnetic resonance imaging (fMRI) study to investigate the blood-oxygenation-level-dependent effect of personalized circumscribed interest rewards versus social rewards in 39 youth with ASD relative to 22 TDC. To probe the reward system, we employed short video clips as reinforcement in an instrumental incentive delay task. This optimization increased the task’s ecological validity compared to still pictures that are often used in this line of research.

**Results:**

Compared to TDCs, youth with ASD had stronger reward system responses for CIs mostly within the non-social realm (e.g., video games) than social rewards (e.g., approval). Additionally, this imbalance within the caudate nucleus’ responsiveness was related to greater social impairment.

**Conclusions:**

The current data support the idea of reward system dysfunction that may contribute to enhanced motivation for RRBIs in ASD, accompanied by diminished motivation for social engagement. If a dysregulated reward system indeed supports the emergence and maintenance of social and non-social symptoms of ASD, then strategically targeting the reward system in future treatment endeavors may allow for more efficacious treatment practices that help improve outcomes for individuals with ASD and their families.

**Electronic supplementary material:**

The online version of this article (10.1186/s13229-018-0195-7) contains supplementary material, which is available to authorized users.

## Background

Neurobiological research in autism spectrum disorders (ASD) has largely focused on social communication impairments, with much less attention on brain mechanisms that cause and maintain restricted and repetitive behaviors and interests (RRBIs) [1]. There is accumulating evidence indicating that both symptom clusters might be mediated, in part, by the same mesocorticolimbic system subserving reward-driven, motivational behaviors [2]. But the reward system’s role in both symptom clusters has not been systematically studied in a single sample of youth with ASD to date.

The social motivation hypothesis postulates ASD as a motivation disorder with affected persons preferring to explore and learn from the non-social environment at the expense of the social world [3]. The imbalance between motivation for social versus non-social stimuli is reflected in the responsiveness of the brain’s reward system [4]. Specifically, this hypothesis posits that some RRBIs may originate, in part, from the reward system being hyper-reactive for circumscribed interests (CIs) mostly within the non-social realm (e.g., mechanical and physical aspects of the environment), whereas social impairments may result, in part, from the reward system being hypo-reactive for socially rewarding stimuli and encounters such as social interactions with positive or non-negative affect [3]. Consequently, the developing child with ASD becomes deprived of crucial social learning opportunities, leading to aberrant social skill development and failed specialization of brain regions subserving social information processing [5].

Most recently, functional magnetic resonance imaging (fMRI) studies have started to address the neural system of reward responsiveness as a proxy for motivation functioning in ASD (see for a review, [6]). Although there is accumulating evidence of neural reward processing dysfunction in this population, findings are decidedly mixed, i.e., the direction and specificity of the deviations are inconsistent [2]. Thus, any firm conclusions are premature at this point. Several studies, however, report aberrant blood-oxygen-level-dependent (BOLD) responses of crucial reward regions in individuals with ASD, including ventral and dorsal striatum, ventromedial prefrontal cortex (vmPFC), anterior cingulate cortex (ACC), and orbitofrontal cortex (OFC), in response to both social rewards (e.g., smiling face, approval) and monetary rewards (e.g., gain of 0.50$) [7–11]. Findings of altered reward system responsiveness in ASD have generally been interpreted as potential neural signatures of decreased motivation to seek and appreciate these types of “conventional” desires.

In contrast, the pursuit of RRBIs, particularly CIs, are reported to be a source of pleasure by affected people [12], and the use of RRBIs as reward contingencies in behavioral modification programs has been found to be therapeutically effective [13]. Given the sporadic behavioral studies addressing the rewarding value of RRBIs, it is not surprising that there has only been limited neurobiological research on the reward system’s potential role in RRBIs [1].

Although the reward system may contribute, at least partially, to all forms of RRBIs [4], CIs may make the ideal candidate for investigating neural reward mechanisms of RRBIs—versus social impairments—in ASD: (1) The non-social quality of CIs stand in stark contrast to the diminished social interests of affected people (i.e., CIs usually are non-socially interesting, not shared, and negatively impact interpersonal relations [12]); (2) CIs can be compared to interests of typically developing controls (TDC); TDCs likely exhibit low rates of other RRBIs during the school age years (e.g., stereotypic body movements); and (3) In practical terms, CIs are relatively easier to re-create and measure in the MRI environment than other RRBI symptoms.

To date, two studies have investigated reward system responsiveness for CIs in ASD [14, 15]. Dichter et al. [6] applied a standardized set of stimuli hypothesized to be “autism-specific objects of high interest” (e.g., pictures of trains, computers) as reward outcomes; their presentation was contingent on accurate task performance in a reaction time task. The study revealed diminished ventral striatal activation for monetary reward in adults with ASD, which was accompanied by enhanced activation in vmPFC for autism-specific object rewards. Because Dichter et al. [6] used standardized object images rather than individualized items, it remains unknown whether the participants’ actual interests were covered by the stimulus set used. More importantly, the study did not contrast the interest rewards with social rewards. Thus, the social motivation hypothesis of ASD was tested only indirectly.

Cascio and colleagues [14] used a passive viewing paradigm with personalized CI pictures of each participant with ASD, focusing on youth rather than adults. The authors report heightened BOLD responses in anterior insula as well as mid-dorsal ACC—critical nodes of the “salience network”—when youth with ASD viewed their own versus others’ interests. The use of a passive task that did not require an active response to maximize reward outcome might explain the lack of group differences for CIs in reward circuitries. Reliable between-group activation differences within core reward regions (e.g., striatum) appear to be critically dependent on the requirement for an instrumental response [16].

For the current investigation, we leveraged the strengths of both prior imaging studies. Like Dichter, Felder and colleagues [6], we applied an incentive delay task to assess neural reward responsiveness with fMRI. Here, we chose a blocked design that has previously shown robust reward system activation in youth with vs. without ASD [7]. Existing imaging experiments targeting motivation in ASD relied on static pictures to serve as appetitive stimuli, which are, at best, only weakly rewarding and can fail to elicit motivational processes. Therefore, we employed dynamic stimuli that are perceived as more natural and engaging and, thus, may serve as more vital incentives [17]. To this end, for the current study, we developed a novel set of video clips of social and interest rewards [18]. More specifically, for the CI reward condition, we created individualized video stimuli for each participant based on self- and parent-reported CIs similar to Cascio and colleagues [14].

Thus, the present fMRI study aimed to compare the brain’s reward system responsiveness to individual CI rewards versus social rewards in youth with ASD relative to TDC. We expected enhanced neural signals in participants with ASD in response to their individual CI reward (in particular, in ventral and dorsal striatum as well as in vmPFC, ACC, and OFC), while neural activation would be reduced for social rewards. Additionally, to specifically test predictions derived from the social motivation hypothesis, we explored correlations between differential reward system responsiveness for CI reward versus social reward and ASD symptom severity.

## Methods

### Participants

Sixty-seven youth, ages 8–17 years, were enrolled in this study, including 45 with ASD (without intellectual disability) and 22 TDC. Five participants with ASD did not attempt the scan, and one child with ASD was excluded for extreme hydrocephalus. All participants stayed within our head motion thresholds during the scan (i.e., root mean square < 1.75 mm of maximum displacement and .0175 rad of translation). The final imaging sample comprised 39 youth with ASD and 22 TDCs. All participants had a General Conceptual Ability (GCA) ≥ 75, equivalent to full-scale IQ, as measured by the Differential Ability Scales—Second Edition [19].

Youth with ASD received an expert clinical diagnosis based on Diagnostic and Statistical Manual of Mental Disorders–Fourth Edition–Text Revision criteria (DSM-IV-TR) [20]; the Autism Diagnostic Interview-Revised [21], and the Autism Diagnostic Observation Schedule [22] were used by experienced clinicians to inform diagnostic decisions. Youth with ASD were excluded if parents reported any known genetic, current mood or psychotic disorder, neurological disorder, premature birth (gestational age ≤ 37 weeks), or other significant medical conditions that affects brain functioning. Youth on atypical antipsychotics were excluded. Youth on psychostimulants were asked to withhold on the day of the study (*n* = 5), and youth taking other psychoactive medication were included (selective serotonin reuptake inhibitors: *n* = 10, selective norepinephrine reuptake inhibitors: *n* = 3, alpha2a-agonist: *n* = 3).

TDCs were excluded if parents reported any known genetic, language, learning, neurological, or psychiatric disorder, premature birth, or first- or second-degree relative with ASD. Youth were also excluded if parents reported elevated symptoms on any scale from the Child and Adolescent Symptom Inventory (CASI-4R; [23]. Groups did not differ in age and sex ratio, but the ASD group had a marginally lower GCA than TDCs (Table 1).

**Table 1:** Summary of participant characteristics by diagnostic group

Measure	TDC (*n* = 22)	ASD (*n* = 39)	*p* value*
Age (years) *M*(SD)	12.85 (2.13)	12.58 (2.37)	0.66
Age range	9.08–17.00	8.17–17.58	
VIQ (SS) *M*(SD)	113.5 (15.4)	105.6 (14.4)	0.06
PIQ (SS) *M*(SD)	109.2 (16.2)	101.9 (16.5)	0.10
FSIQ (SS) *M*(SD)	111.9 (18.0)	103.6 (15.7)	0.08
Sex ratio (male:female)	17:5	29:10	0.80
ADOS-2 social affect + RRB	–	11.62 (3.89)	–
ADOS-2 severity	–	6.87 (0.32)	–
ADI-R social Interaction	–	18.39 (5.20)	–
ADI-R communication	–	14.61 (4.87)	–
ADI-R repetitive behaviors	–	6.03 (2.09)	–
RBS-R (total score)	–	17.87 (11.88)	–
SRS-2 (total *T*-score)	39.5 (4.87)	73.41 (10.71)	< 0.001
IS (total score)	9.53 (2.41)	14.2 (3.76)	< 0.001

*VIQ* verbal IQ, *PIQ* performance/nonverbal IQ, *FSIQ* full-scale IQ, *SS* standard score (*M* = 100; SD = 15), *SRS-2* Social Responsiveness Scale—2nd Edition, *ADOS-2* Autism Diagnostic Observation Schedule Module 3 or 4, *ADI-R* Autism Diagnostic Interview—Revised, *RBS-R* Repetitive Behavior Scale-Revised, *IS* Interests Scale

*p values are based on two-sample *t* tests and *χ*^*2*^ test (for sex ratio)

### Phenotypic measures

For this study, we developed the Interest Preference Assessment (IPA), a short interview adapted from the Interests Scale [24, 25], to directly evaluate each child’s most favorite interest/hobby to be used in the fMRI task (see Additional file 1). The IPA is comprised of two sections: The first section consists of a list of 25 categories of interest (e.g., machines or figuring out how things work, animals, people), and children are asked to rate each interest on a scale from 1 to 5, with 5 indicating “I could do this activity or talk about this topic all the time.” The second part of the IPA asks children to identify their primary interest and answer questions assessing the interference and intensity of that interest (e.g., “How much time do you spend doing or thinking about this interest or hobby? Does this get in the way of other responsibilities? Do you get annoyed or upset when you are asked to stop talking or doing this interest or hobby?”). Most children identified a singular primary interest, but a few children identified more than one interest as their “favorite.” In these cases, we asked them to pinpoint the interest they would most enjoy having in the upcoming MRI session. Examples of interests include videogames, professional sport teams, musicians/actors, toys, elevators, and movies (Additional file 1: Table S1).

Additionally, all parents were asked to complete the Social Responsiveness Scale-2nd Edition (SRS-2; [26]), the Repetitive Behavior Scale-Revised (RBS-R; [27, 28]), and the Interests Scale (IS; [24, 25]) to dimensionally assess behaviors characteristic of ASD.

Participants were compensated for their participation in this study. Written informed consent was obtained from all participants and their parents. This study was approved by the local Institutional Review Board.

### FMRI task

**Fig. 1:**
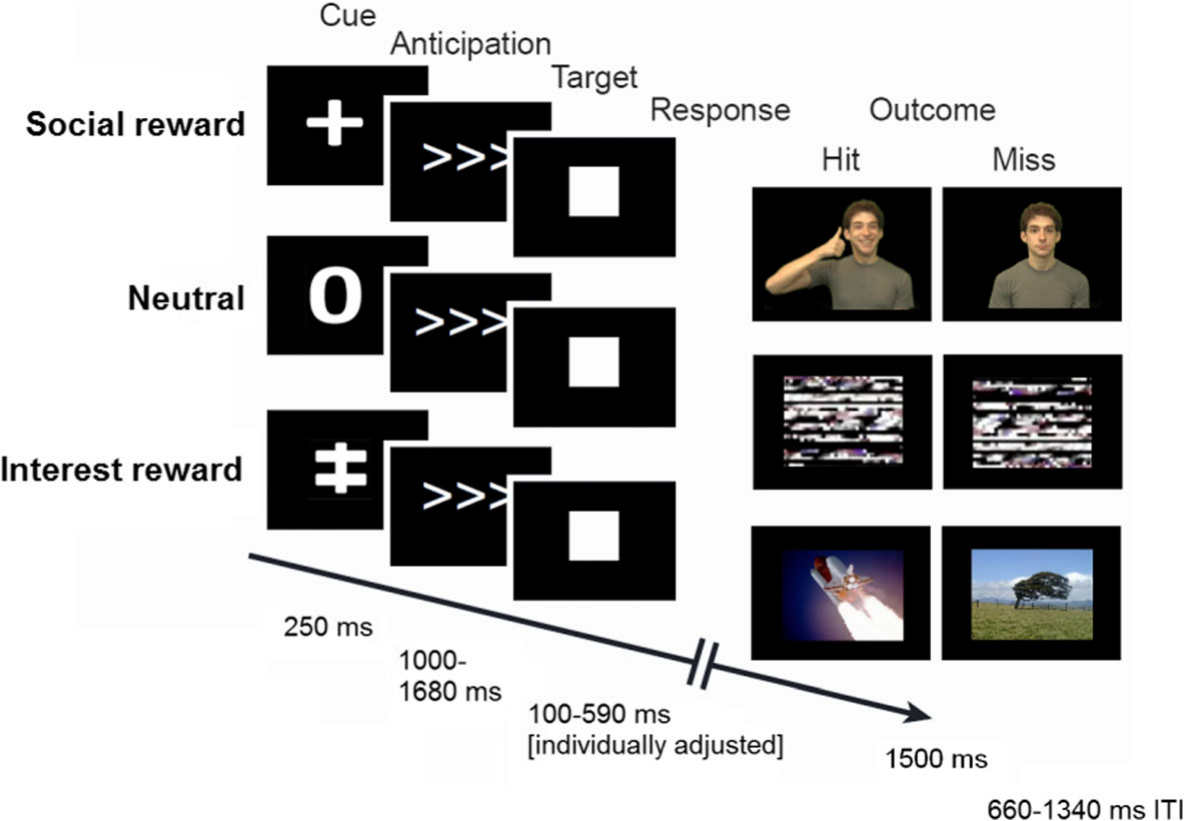
Illustration of the incentive delay task in a blocked fMRI design, including three different reward conditions: social reward (SR), interest reward (IR), and neutral (NR). To increase the ecological validity of the paradigm, static photos were replaced with short video clips (see text for more details)

We used an incentive delay task (IDT) in a blocked fMRI design that is an adaptation of the “classic” IDT [29] and aims to examine participants’ motivation to receive either a social or a personalized type of reward based on individual interests (vs. neutral outcome). To maximize ecological validity, we utilized short movie clips of actors expressing facial expressions along with other nonverbal gestures in the social reward condition as well as short video clips depicting personalized interests in the interest reward condition (Fig. 1 and Additional file 1 for details).

The IDT is a simple speeded button press task to examine neural responses to rewards that can be gained dependent upon a person’s ability to quickly and accurately respond to a target symbol in each trial. An online tracking algorithm was implemented to continuously monitor and adjust the target duration according to individual performance to achieve an accuracy rate of ≥ 50% (see Table 2). In social reward (SR) trials, target hits resulted in a short video clip of a person showing happy facial expressions, including nodding with a smile and a praising nonverbal gesture—the “thumbs up” sign. The outcome for misses was a video clip of a person showing neutral expressions, with slight natural motion (e.g., eye blinks). In personalized interest reward (IR) trials, target hits resulted in a short video clip of a person’s favorite interest (e.g., Minecraft©), whereas the outcome for misses was a video clip of a tree with slight natural movement (i.e., breeze gently moves leaves). The outcome in the neutral (NR) condition was always a video clip of “TV static.” The SR video clips portrayed six different adult actors (half female, half male, of various races and ethnicities representative of the local region) and six unique clips of personalized interests were presented in the IR condition; each of which was randomly repeated eight times throughout the experiment. Actor’s videos were chosen from a larger pool based on ratings of the actor’s likability as well as based on her authenticity of depicting approval; only actors who had the highest ratings on both scales were included (see [18]). Interest clips were chosen from various youtube© videos to depict the participants’ individual interests. All video clips fulfilled the following criteria: HD quality, multicolored content, disabled audio, .avi format, resolution 720 × 400 pixels, 25 frames/s, and 1500 ms duration; and the actor or interest/hobby is depicted in the center of the screen.

**Table 2:** Main performance variables of the incentive delay task by group and incentive condition

	TDC *M*(SD)	ASD *M*(SD)	*p* value*
RT for hits (in ms)			
Neutral	255.3 (29.8)	250.9 (27.7)	0.56
Social reward	206.7 (22.4)	200.7 (20.0)	0.29
Interest reward	242.4 (23.9)	238.4 (28.3)	0.58
Accuracy (in %):			
Neutral	49.5 (11.7)	47.7 (9.9)	0.53
Social reward	56.9 (9.9)	54.8 (6.7)	0.32
Interest reward	45.1 (11.6)	45.7 (7.6)	0.83

*p values are based on two-sample *t* tests

There were two 6 min 44 s runs that presented the three experimental conditions (i.e., SR, NR, and IR) block-wise, with a fixation period interspersed after each block. Altogether, 6 blocks per condition and 18 fixation periods were presented across the two runs. Within each run, trial types were designated using intuitive cues signaling the reward type that could be obtained in the ongoing trial for correct performance: a plus sign for SR trials, the numeral zero for NR trials, and a double-plus sign for IR trials. Each block consisted of a total of 7 trials across a total of 3 presentations per run (21 trials per run and 42 trials per condition across both runs). Each trial started with a condition cue for 250 ms, followed by a variable anticipation phase (three arrows were displayed in the middle of the screen; 1000–1669 ms) and the target appearance (white solid square; individually tailored duration of 100–590 ms based on the online tracking algorithm). Feedback was presented immediately after target disappearance for a duration of 1500 ms, followed by a jittered inter-trial interval (660–1340 ms). Jittering was optimized so that each trial lasted exactly two TRs (4.68 s). Block duration was 32.76 s. The fixation period between each block flashed a crosshair in each corner of the screen and then the center of the screen for the duration of a single TR (2.34 s × 5 crosshairs = 11.7 s).

To ensure that all participants fully understood the different cue-outcome relations, i.e., to avoid a learning component during the experiment, each participant received training right before the scan. This was followed by a performance-based test of their understanding as well as a practice session of the task. Only participants who fully understood the task instructions (i.e., remembered each cue-outcome relation in all three reward conditions for hit and miss trials) moved on to the practice session of the task, followed by the real fMRI scan. All participants passed the test with 100% accuracy.

### Image acquisition

All imaging data were collected using a Siemens Verio 3T scanner (Erlangen, Germany) and a 32 multichannel headcoil. Functional data consisted of two 6-min 44 s runs of whole-brain T2*-weighted BOLD echo planar images with 173 volumes acquired per run, including two “dummy” scans at run onset allowing for T1 magnetic saturation (40 oblique axial slices, isotropic voxel size = 3.5 mm, TR/TE = 2340 ms/25 ms, flip angle = 60°). Two high-resolution structural MR images were acquired for the registration of fMRI data to MNI space: A T1-weighted MPRAGE sequence of the entire brain (176 sagittal slices, isotropic voxel size = 1 mm, TR/TE = 1900 ms/2.54 ms, flip angle = 9°) and a FLASH sequence collected in the same plane as the fMRI data (number of slices = 40, slice thickness = 3.5 mm, TR/TE = 300 ms/2.46 ms, flip angle = 60°).

### Image analysis

Functional image processing and statistical analyses were then carried out using FEAT (FMRIB’s Expert Analysis Tool), part of FMRIB’s Software Library (FSL) package [30, 31]. Prior to image analysis, the first two images of the functional data set were discarded because of the non-equilibrium state of magnetization. Each time series was despiked using AFNI’s 3ddespike program, motion-corrected, temporally filtered (nonlinear high-pass filter with a 1/90 HZ cutoff calculated with FSL’s cutoffcalc), and a 3D Gaussian filter (FWHM = 5 mm) was applied to account for individual differences in morphology and local variations in noise. Voxel-wise regression analyses were performed on each of the participant’s runs using FILM (FMRIB’s Improved Linear Model). Motion parameters (i.e., six parameters corresponding to three directions of translation and three axes of rotation) were entered as nuisance regressors (absolute mean displacement: ASD = 0.13 ± 0.05 mm, TDC = 0.14 ± 0.04 mm, *p* = 0.38; relative mean displacement: ASD = 0.09 ± 0.04 mm, TDC = 0.10 ± 0.04 mm, *p* = 0.79). Each task condition (SR, IR, and NR) was coded as an explanatory variable (EV) and convolved with a double gamma function, along with its temporal derivative. Each EV yielded a per-voxel parameter estimate (*β* map) that represented the activation magnitude associated with that regressor. In order to create comparisons of interest, *β* maps were contrasted. Functional data were registered to MNI stereotactic space using affine transformations. Next, within-subject analyses across runs employed a fixed-effects model, whereas group-level inferential statistical analyses were carried out on each contrast of interest (e.g., IR vs. SR, IR vs. NR, and SR vs. NR) using FMIRB’s linear analysis of mixed effects (FLAME1+2), followed by two-sample *t* tests (TDC vs. ASD). Whole-brain *Z*-statistic (Gaussianized *T*) maps were thresholded using clusters determined by a voxel level of *Z* ≥ 3.1 (i.e., *p* ≤ 0.001) and an FWE-corrected cluster-significance threshold of *p* ≤ 0.05 to strictly control type I errors [32]. In addition, to test our a priori hypothesis of greater reward system activation for individual CI reward than social reward in ASD vs. TDC, we examined group differences in six regions of interest (ROI), including ventral striatum/nucleus accumbens (Nacc), dorsal striatum/caudate, ACC, vmPFC, OFC, and insula, which were anatomically defined areas from the Harvard-Oxford structural probabilistic atlases [33]. We applied a FWE-corrected threshold of *p* ≤ 0.05 across each region using the Randomise v2.1 program as part of FSL [34].

## Results

### Behavioral task performance

Reaction times (RTs) for hits (in ms) and task accuracy (correct response rate in %) on the IDT were analyzed using a three (reward: NR vs. SR vs. IR) by two (group: ASD vs. TDC) repeated-measures MANOVA model, followed by planned contrasts. This analysis revealed a main effect of reward [*F*(4,56) = 143.65, *p* < 0.001, Cohen’s *d* > 1.4], but no main effect of group [*F*(2,58) = 0.53, ns] or group-by-reward interaction effect [*F*(4,56) = 1.13, ns]. This suggests that the different reward conditions similarly affected behavioral performance across groups. The following univariate ANOVAs showed that the significant reward effect was related to both speed (*p* < 0.001, Cohen’s *d* > 1.4) and accuracy (*p* < 0.001, Cohen’s *d* > 1.4). Regarding RT, post hoc contrasts revealed fastest RTs for SR, slowest RTs for NR and with the IR condition intermediate (all *p*s < 0.001, all Cohen’s *d*s > 1.06). This indicates that incentive manipulations within the experimental task were successful. Post hoc contrasts for accuracy revealed the highest the correct response rate for SR, the lowest the correct response rate for IR and the NR condition intermediate (all *p*s < 0.004, all Cohen’s *d*s > 0.77) (see Table 2). Although we intended to maintain an equal average accuracy across conditions and participants by adjusting the target duration according to individual trial-by-trial performance, our finding of different accuracy rates for the three incentive conditions is in line with prior studies [11].

### Reward system responsiveness across both study groups

**Fig. 2:**
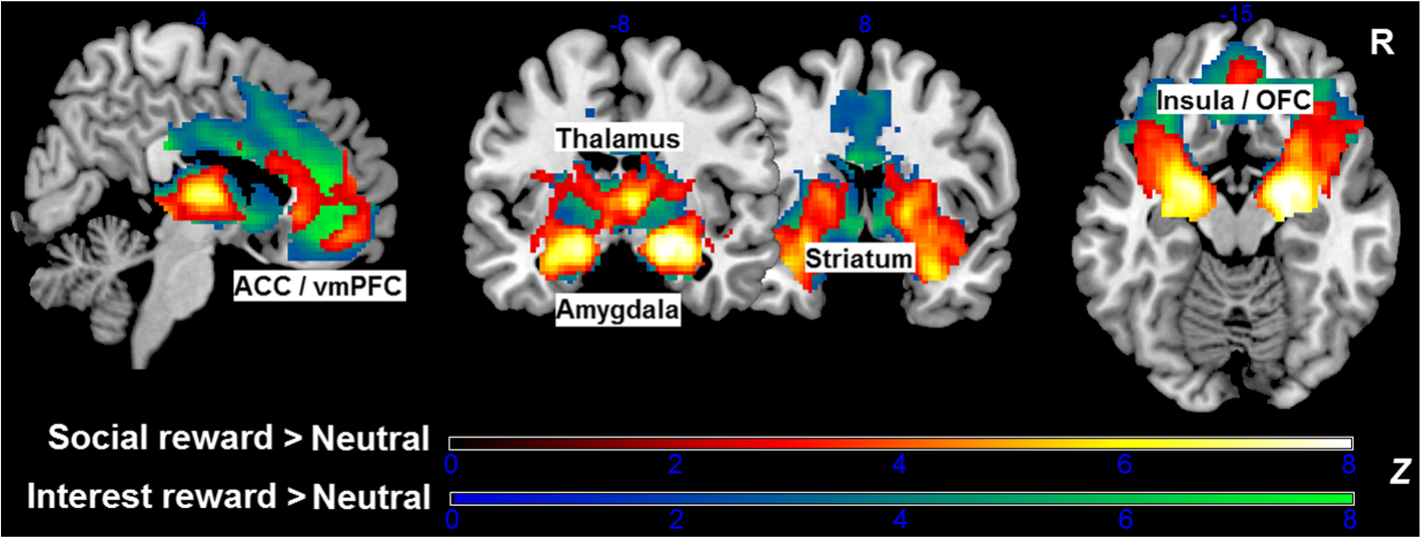
*Z*-statistic activation maps depict reward system activation across the entire sample separately for the two high-level contrasts social reward > neutral outcome (hot colors) and interest reward > neutral outcome (cool colors). Both social reward and interest reward (versus neutral outcome) strongly activated a widespread reward system, comprising ventral and dorsal striatum, thalamus, amygdala, medial prefrontal areas (ACC, vmPFC) as well as clusters with orbitofrontal cortex (OFC) and anterior insula. Maps were thresholded using clusters determined by a voxel-level of *p* ≤ 0.001 and an FWE-corrected cluster-significance threshold of *p* ≤ 0.05. Color bars indicate *Z*-scores

Considering reward system responsiveness across both groups for the two high-level contrasts, i.e., SR > NR and IR > NR, the whole-brain analysis revealed robust brain activation (i.e., *k* ≥ 10) in ventral striatum (including Nacc), dorsal striatum (including caudate nucleus, and putamen), thalamus, amygdala, ACC, vmPFC, insula, and OFC (Fig. 2). Considering differential reward system activation for the two reward types, the IR > SR comparison showed greater BOLD responses in a cluster, comprising Nacc, caudate, thalamus, ACC, vmPFC, insula and OFC (MNI_peak_ = − 2, 42, 0; *Z*_max_ = 9.16; *k* = 8047) as well as a cluster within posterior cingulate cortex (MNI_peak_ = 0, − 30, 28; *Z*_max_ = 9.00; *k* = 166). The reversed comparison (i.e., SR > IR) yielded significant activation differences within right and left insula (MNI_right-peak_ = 36, − 18, 20; *Z*_max_ = 5.18; *k* = 802, and MNI_left-peak_ = − 42, − 18, 6; *Z*_max_ = 4.87; *k* = 500).

### Reward system imbalance for interest reward versus social reward in ASD

**Fig. 3:**
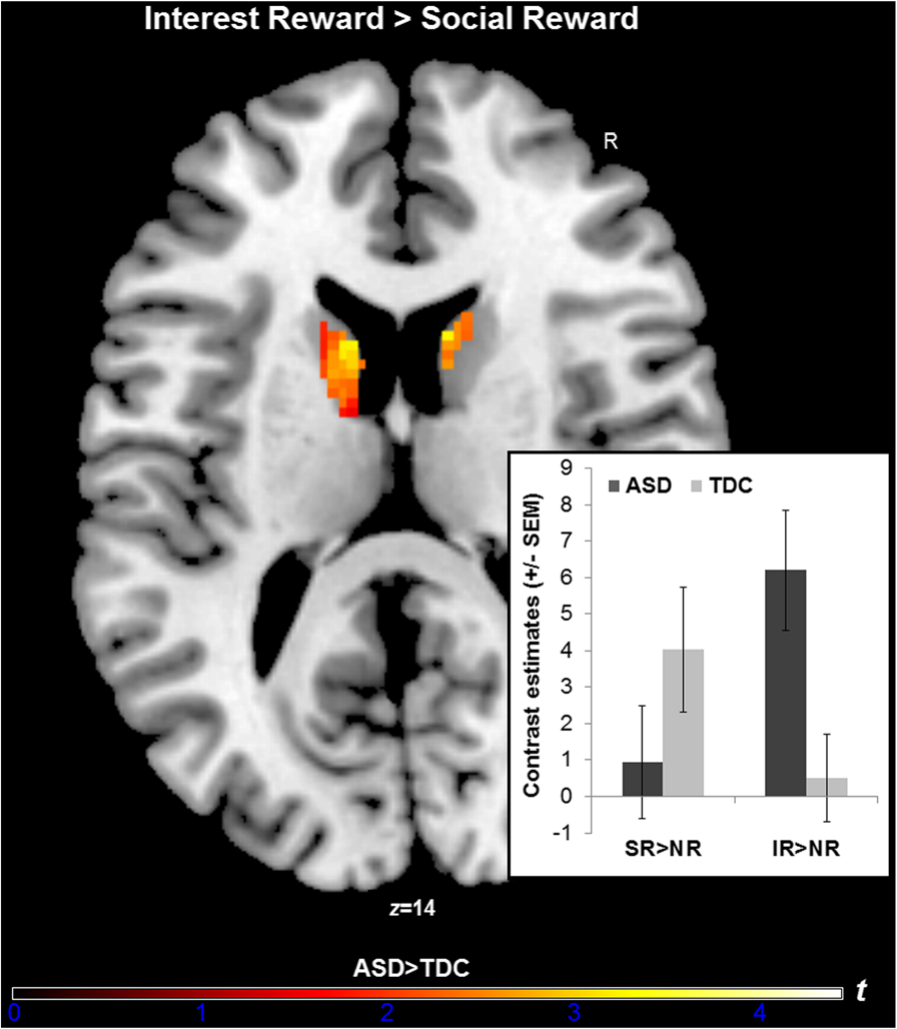
ROI analyses revealed a significant group-by-reward type interaction effect in the caudate nucleus with greater dorsal striatum activation for interest reward (IR) than social reward (SR) in youth with ASD, while TDCs did not show significant differences—although a reverse direction—in their response pattern to both reward types. Bar graphs depict mean β values from the bilateral caudate cluster identified by the significant interaction effect. The ROI was anatomically defined based on the Harvard-Oxford structural probabilistic atlas. Results are FEW corrected at *p* ≤ 0.05 across this particular region. Color bar indicates t-statistics

Following up our hypothesis of reward “imbalance” in ASD (i.e., enhanced reward system activation for CI reward, but diminished responsiveness for social reward), we specifically investigated group activation differences in response to IR versus SR. Using whole-brain cluster thresholding that strictly controls type I errors [32], neither the IR > SR contrast nor the SR > IR contrast revealed significant group activation differences. The additional ROI analyses, however, demonstrated that the right caudate (MNI_peak_ = 12, 14, 14; *t*_max_ = 3.14; Cohen’s *d* = 0.84; *k* = 26) as well as the left caudate (MNI_peak_ = − 12, 6, 12; *t*_max_ = 3.14; Cohen’s *d* = 0.89; *k* = 96) were more active for IR than SR in youth with ASD relative to TDC; or put differently, the caudate was less active to SR than IR in ASD versus TDC (Fig. 3). None of the other a priori ROIs (i.e., Nacc, vmPFC, ACC, OFC, and insula) revealed significant group differences for IR > SR and SR > IR.

To further examine the differential caudate response, we extracted individual *β* values from this bilateral cluster, separately for group and reward type (see lower right of Fig. 3). The repeated-measures ANOVA performed on the *β* values as the dependent variable confirmed the significant group-by-reward interaction effect [*F*(1,59) = 10.46, *p* = 0.001, Cohen’s *d* = 0.84]; main effects of reward and group were non-significant (*p*s > 0.51). Follow-up within-group *t* tests yielded a significantly greater caudate response for interest reward than social reward in the ASD group [*t*(38) = − 3.05, *p* = 0.004, Cohen’s *d* = 0.53], while TDC showed marginally stronger caudate activation for social reward compared to interest reward [*t*(21) = 1.83, *p* = 0.081, Cohen’s *d* = 0.51]. Between-group *t* tests yielded significantly stronger caudate activation for IR in the ASD group compared to TDC [*t*(59) = 2.79, *p* = 0.007, Cohen’s *d* = 0.74], but no significant activation differences between groups in response to SR within this cluster [*t*(59) = − 1.28, *ns*, Cohen’s *d* = 0.34]. The group-by-reward interaction effect remained significant when age and GCA (i.e., FSIQ) were controlled for (*p* < 0.001, Cohen’s *d* = 0.93).

Given the nonselective nature of this analysis [35], we additionally investigated unbiased individual *β* values averaged across an anatomically defined bilateral caudate mask (based on the Harvard-Oxford probabilistic atlas). We found that the group-by-reward interaction effect was still significant, but with a lower, though still medium strong, effect size [*F*(1,59) = 4.34, *p* = 0.04, Cohen’s *d* = 0.55]. Please also note that there were no significant between-group differences in brain activation for the neutral condition using whole-brain or ROI analyses (NR > fixation for structurally defined bilateral caudate: *t*(59) = − 1.04, *ns*).

Because our study sample comprised of substantially more youth with ASD than TDC, we repeated our structural ROI analyses for right and left caudate in an age- and IQ-matched sub-sample of 22 individuals with ASD (14 males, 8 females; age 12.3 ± 2.4 years; IQ 110.2 ± 17.2) and the initial group of 22 TDC (17 males, 5 females; age 12.9 ± 2.1 years; IQ 111.9 ± 18.0). The results remained virtually the same, with significantly greater bilateral caudate activation for IR than SR in the 22 youth with ASD relative to the 22 TDC (right MNI_peak_ = 10, 14, 14; *t*_max_ = 3.55, left MNI_peak_ = − 12, 6, 12; *t*_max_ = 3.24). Taken together, these results suggest that the caudate nucleus responded differently in ASD versus TDC dependent on the reward type. This effect was driven by particularly greater dorsal striatum responsiveness for personalized interest reward than social reward in ASD, while TDCs did not show significant differences—although a reverse direction—in their response pattern to both reward types.

### Correlations between caudate response, clinical symptom severity, and RTs

**Fig. 4:**
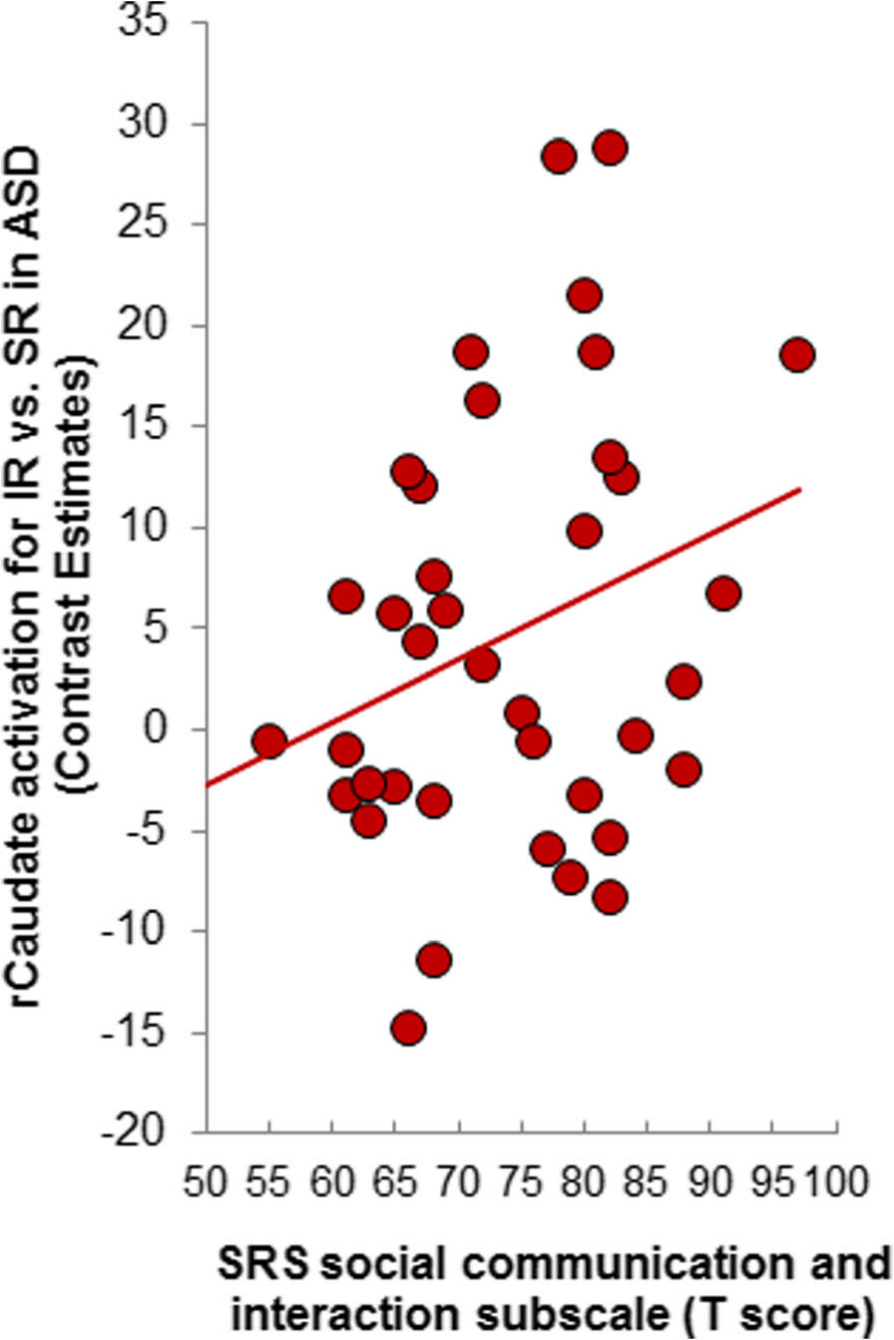
The magnitude of caudate activation that distinguished the two groups correlated positively with ASD symptom severity as assessed by the SRS-2 social communication and interaction sub-score (for the ASD group only). The stronger the caudate activation for personalized interest reward vs. social reward the greater the social impairment in the ASD group

To specifically test predictions derived from the social motivation hypothesis, we correlated the individual *β* values from the cluster that distinguished the two groups (i.e., magnitude of caudate response for IR > SR) with the degree of social dysfunction in ASD as assessed by the autism diagnostic observation scale (ADOS) severity score [36] and the SRS-2 social communication and interaction (SCI) subscale’s *T*-score [26]. This analysis yielded a significant positive correlation between (right) caudate responses and SRS-2 SCI scores (*r*(39) = 0.31, *p* = 0.05; ADOS: Spearman’s *ρ*(39) = − 0.02, *ns*), such that stronger caudate activation for interest reward than social reward was related to greater social impairment in the ASD group (Fig. 4). Because of our a priori hypothesis, we did not apply corrections for multiple comparisons.

We further explored associations between bilateral caudate responses for IR > SR and RRBI symptoms in ASD as assessed with the RBS-R (total score), IS (total score), ADI-R (RRB total score), ADOS-2 (RRB total score), and SRS-2 (restricted interests and repetitive behavior subscale *T*-score). This analysis revealed a significant positive correlation with ADOS-2 RRB symptom expression (Spearman’s *ρ*(39) = 0.41, *p* = 0.011; Additional file 1: Table S2), such that stronger caudate responsiveness for CI reward than social reward was associated with greater (clinically observed) RRBIs in our ASD sample. This correlation, however, would not survive correction for multiple comparisons.

We additionally found a significant negative correlation between (left) caudate activation differences to IR > NR and ΔRT for IR vs.NR in the ASD group (*r*(39) = − 0.32, *p* = 0.05). This suggests that stronger caudate responsiveness to CIs versus neutral outcome was associated with faster behavioral responses by the participants with ASD to attain this individual reward. Please note, though, that this correlation would not survive correction for multiple comparisons.

## Discussion

The results of this fMRI study are in line with predictions by the social motivation hypothesis [3, 37]: Compared to TDCs, youth with ASD had stronger reward system responses for CIs mostly within the non-social realm (e.g., video games) than social rewards (e.g., approval). Additionally, aberrant reward system responses—most pronounced within the caudate nucleus-were related to greater social impairment. Behavioral task performance did not differ between the two study groups, emphasizing the power of neurobiological data in revealing atypical motivational mechanisms in ASD and at the same time avoiding a potential confounding factor [38].

The current investigation advanced the strengths of the two preceding imaging studies targeting CIs in ASD [14, 15] by (a) employing a newly created set of video clips as reinforcement in (b) a well-established instrumental reward task to (c) reliably contrast reward system responsiveness for individual CIs versus social approval rewards. Thus, we tested the social motivation hypothesis of ASD more directly, and we applied more ecologically valid reward stimuli. Using a standard set of static images hypothesized to be “autism-specific objects of high interest” versus monetary reward as incentives, Dichter and colleagues [6] previously reported blunted striatal responses to both reward types in adults with ASD, accompanied by enhanced vmPFC activation for object rewards. Standardized objects as a proxy for CIs, however, provide a potentially weaker estimate of the neural reward effects that could be identified with individualized CI items. Moreover, instead of simple static stimuli, we utilized, for the first time in this line of research, dynamic incentives that are commonly experienced as more naturalistic and appealing and, thus, may elicit both stronger and more reliable motivational responses [39].

The aberrant pattern of caudate activation in ASD not only supports prior research but also highlights a potentially fundamental difference in how individuals with ASD prioritize CI stimuli over social stimuli. Our finding of greater BOLD responses for CI rewards than social rewards in youth with ASD compared to a more balanced response pattern in TDCs substantiates the earlier report by Dichter and colleagues [6]. In contrast to our hypothesis, our follow-up analyses did not reveal specifically diminished caudate responses to social reward when directly comparing the two groups, at least with the social incentives used in this study sample. Other recent functional imaging studies, however, did reveal aberrant caudate responses to both social and monetary reward in ASD [7, 11]. Converging evidence from human and nonhuman research links the caudate nucleus directly to decision-making processes, specifically to the selection and initiation of purposeful actions in order to maximize reward outcome [40]. In this regard, we found that greater BOLD responses in the left caudate to CI rewards were associated with faster reaction times by the participants with ASD to obtain this positive outcome (but only at an uncorrected significance level). This suggests that in youth with ASD the caudate nucleus may optimize goal-directed actions for CIs to a greater extent than for social rewards or other types of “conventional” desires (e.g., money). Whether the caudate nucleus is also involved in aberrant instrumental responsiveness to primary rewards, such as food [41], should be investigated in future studies.

Notably, we detected that stronger caudate responsiveness for CIs (versus social reward) was related to greater overall symptom severity among our participants with ASD. Cascio and colleagues [14] recently reported that activation of the insula—as part of the “salience network”—in response to viewing pictures of one’s own vs. others’ interests was positively correlated with CI severity, but not overall clinical symptomatology, in youth with ASD. As noted in the Introduction, an instrumental task with active response-outcome contingencies is critical for stimulating reward pathways [16]. A passive viewing task may be less optimal for observing reward system responsiveness in relation to ASD symptoms. Thus, our findings emphasize the connection between the caudate nucleus as a core reward region involved in goal-directed behavior and the clinical phenotype of ASD (see also [42]).

Although several plausible models have been advanced to explain RRBIs in ASD (e.g., inhibitory control deficit), the relatively overactive caudate nucleus for CIs, as found in the present study, supports the idea that ASD may be in part a motivational disorder of “behavioral dependency” to RRBIs because of the rewarding effects they induce [4]. Different authors have argued that the rewarding effect of RRBIs, including CIs, may originate from the need of people with ASD for predictability, i.e., lawful and deterministic events in their environment, where they can exert more control [43]; rapid and uncertain moment-by-moment changes of a person’s behavior inherent in social encounters are the opposite. The unpredictable nature of social encounters may make them unrewarding or even anxiety-provoking and aversive for individuals with ASD [44]. RRBIs, such as CIs, in turn, may offer a pleasurable compensation for the unpredictable social world.

The exact mechanisms as to how the dominating reward effects of CIs emerge and interfere with the reward value of social engagement in ASD are, however, yet unclear. When CIs are indeed rewarding, their pursuit may be strengthened through self-reinforcement that turns them into rigid habits [45]. The self-reinforcing character of RRBIs, like CIs, may hijack the normal developmental trajectories of entire repertoires of behaviors, including social ones. We suggest that the caudate nucleus—in concert with other frontolimbic structures [46]—may dominate the formation and maintenance of RRBIs. On a daily basis, RRBIs hinder social development and functioning because they absorb resources typically dedicated to social learning opportunities [4]. This view converges with recent evidence demonstrating the role of the caudate nucleus, as part of a cortico-striatal-thalamo-cortical loop [47], in CIs as well as compulsive and ritualistic behaviors in individuals with ASD across different age groups [42, 48–51].

While we could demonstrate robust neural activation differences between ASD and TDC for CI rewards versus social rewards in dorsal striatum, other brain regions that have previously been related to CIs did not emerge in our study (i.e., ventral striatum/Nacc and vmPFC [15] as well as ACC and insula [14]). Despite our specific finding of an imbalance of caudate nucleus responsiveness in ASD, we acknowledge that CIs are not limited to a single anatomical correlate, but are mediated by various distinct yet interacting subcortical and cortical systems [1, 47]. Follow-up studies are warranted to better define how different subcomponents of reward processing—and their neural correlates—contribute to the emergence of and adherence to CIs. Incentive delay tasks, such as the one applied here, can principally be used to test reward learning, reward anticipation and reward valuation as three crucial reward components to consider with regard to CIs and the responsiveness to other “conventional” rewards in ASD [2]. While each reward component has been associated with some distinct (and some interrelated) neural correlates [52], recent human research highlights, however, the difficulty to effectively decompose the neural signals of the various components within a single experimental paradigm (see for a discussion on this issue, [53]). Thus, we foresee that refined inventories of specifically tailored reward measures—preferably grounded in preclinical studies and adequately validated in humans—will benefit this line of research.

Importantly, future fMRI investigations need to control more strictly for multiple comparisons at the whole-brain level. This will help avoiding high degrees of false positive findings and, thus, will ensure that neuroimaging results—particularly between-group findings—are more reliably reproduced. In the present study, we implemented most recent recommendations from the literature using whole-brain cluster thresholding that rigorously controls type I errors [32]; this could also explain why we were not able to replicate some of the previous findings (see Additional file 1: Table S3 for prior analytic approaches in this line of research).

This study had the following limitations: While we presented individually tailored interest clips, we did not utilize personalized social rewards, such as approval clips by caregivers, or preferred peers. Several recent investigations, however, indicate that personally meaningful social incentives hold similar reward value for youth with ASD as unfamiliar social rewards (e.g., [54, 55]); thus, it is unlikely that this potential confound fully accounts for the present findings. An imminent drawback to the use of unique interest items was their diversity among participants, which possibly added noise into the data. We endeavored to strictly equate stimulus properties across participants and conditions. Stimulus diversity (including differential complexity and luminance of video materials), however, may have precluded us from having enough statistical power to reveal additional regions involved in core reward mechanisms of CIs due to BOLD signal heterogeneity (e.g., Nacc, or vmPFC). Moreover, using a blocked design did not allow parsing brain responses for reward learning, anticipation, and consumption. Finally, because we explicitly targeted CIs, our findings are limited to this specific type of RRBI. A logical next step would be to extend this line of research to elucidate reward functions with regard to stereotypies, insistence on sameness, and sensory responses (e.g., [56]) using fMRI as well as other imaging methods, such as event-related brain potentials [57].

## Conclusion

In conclusion, the results of this fMRI study add to the emerging clinical and imaging evidence of striatal involvement in the ASD pathophysiology [58]. More precisely, we demonstrated aberrant caudate nucleus responsiveness in ASD, with greater brain responses for CIs relative to social reward that was also associated with social impairment. We did not find, however, specifically diminished reward system responses to social reward when directly comparing youth with ASD to TDC, at least with the social rewards (i.e., approval from unfamiliar adults) used in the present study. We speculate that reward system dysfunction—most pronounced within the caudate nucleus—may contribute to enhanced motivation for RRBIs in ASD, accompanied by diminished motivation for social engagement. If a dysregulated reward system indeed supports the emergence and maintenance of social and non-social symptoms of ASD, then strategically targeting the role of reward mechanisms will allow for the development of more efficacious treatment practices to better support individuals with ASD and their families.

## Additional file

Additional file 1: Additional methods and tables. (DOCX 48 kb)

## Abbreviations

ACC: Anterior cingulate cortex
ADI-R: Autism Diagnostic Interview-Revised
ADOS: Autism Diagnostic Observation Schedule
ASD: Autism spectrum disorder
avi: Audio video interleave
BOLD: Blood-oxygen-level-dependent
CASI: Child and Adolescent Symptom Inventory
CI: Circumscribed interest
DSM-IV-TR: Diagnostic and Statistical Manual of Mental Disorders–Fourth Edition–Text Revision
EV: Explanatory variable
fMRI: Functional magnetic resonance imaging
FSIQ: Full-scale intelligence quotient
FWE: Family-wise error rate
GCA: General conceptual ability
HD: High definition
IDT: Incentive delay task
IPA: Interest Preference Assessment
IQ: Intelligence quotient
IR: Interest reward
IS: Interests Scale
Nacc: Nucleus accumbens
NR: Neutral
OFC: Orbitofrontal cortex
PIQ: Performance/nonverbal IQ
R: Social reward
RBS-R: Repetitive Behavior Scale-Revised
ROI: Region of interest
RRBI: Restricted and repetitive behaviors and interests
RT: Reaction time
SRS-2: Social Responsiveness Scale—2nd Edition
SS: Standard score
TDC: Typically developing controls
VIQ: Verbal IQ
vmPFC: Ventromedial prefrontal cortex
