# Supplementary material for: Altered reward system reactivity for personalized circumscribed interests in autism
Source: Mol Autism. 2018 Jan 30;9:9. doi: 10.1186/s13229-018-0195-7 (PMC5791309; doi:10.1186/s13229-018-0195-7)
Supplement: Additional file 1: — Additional methods and tables. (DOCX 48 kb) [file 13229_2018_195_MOESM1_ESM.docx]

**Example items of the Interest Preference Assessment (IPA)**

| **Please rate how much you like each interest on a scale from 1 to 5.**  1 = “I am not interested in this topic or activity at all. I would not do it.”  2  3 = ”I am interested in this topic or activity, but it’s not my favorite thing.”  4  5 = “I could do this activity or talk about this topic all the time.”  ***ONLY for interests or activities rated as a 4 or 5:** Please choose something in each category. If you like a category but your favorite thing is not listed, then please write it in on the blank line.   1. How much do you like **machines or figuring out how things work**? ____________    1. Which of these do you like the most?       1. Watches       2. Vacuums       3. ___________________________   […]  9) How much do you like **games**? ____________   - 1. Which of these do you like most?      1. Videogames, board games, card games by myself      2. Videogames with friends (in person)      3. Videogames with friends (online)      4. Board games or card games with friends      5. ___________________________   […] |
| --- |

**FMRI reward task instructions and training procedure**

First, the experimenter showed the training slides and explained the task on a laptop:

*“For this game, we need you to learn three different ‘get ready signals’. The first get ready signal is a PLUS sign. When you see the PLUS sign, get ready. Then, after a couple of seconds, you will see a white square. When you see the white square, press the button as quickly as you can. If you press the button quickly enough, then you will see someone giving you a smile and a ‘thumbs up’. However, when you see the plus sign and don't press the button in time, you will just see someone looking at you. Does that make sense? The second get ready signal is a DOUBLE PLUS sign. If you press the button quickly enough, you will see a short video clip of something that you are interested in. If you press the button before you see the white square or too long after, you will see a video of a tree. Do you have any questions about that? The third get ready signal is a ZERO. After you see the zero, you will see the white square, and, again, we want you to press the button as quickly as you can. But this time, you will always see a video of lines moving on the screen. You won't know how you did. So, when you see the plus sign or the double plus sign, you'll find out how well you did, but when you see the zero, you won't find out how well you did. Does that make sense?”*

Participants were then tested for their understanding of the task instructions:

*“Before we move on, I would like to see whether you remember the rules of the games.*

*Let’s start with the ZERO sign.*

*After you see the ZERO and press the button, what will you see?*

*(Correct answer: The moving lines.)*

*If you see the PLUS sign, and DO press the response button in time, what will you see?*

*(Correct answer: The person showing ‘thumbs up’.)*

*If you see the PLUS sign and DO NOT press the button in time, what will you see?*

*(Correct answer: The person just looking back at you.)*

*Now let's talk about the DOUBLE PLUS sign.*

*If you see the DOUBLE PLUS sign, and DO press the response button in time, what will you see?*

*(Correct answer: Your interest (example is rocket ship))*

*If you see the DOUBLE PLUS sign and DO NOT press the button in time, what will you see?*

*(Correct answer: The tree.)*

Only participants who completely understood the task instructions (i.e., remembered each cue-outcome associations for hit and miss trials) moved on to the practice session of the task. Participants performed several blocks of the task on a laptop outside the scanner. The task was aborted when the participant had played at least one block of each condition.

Participants were then brought to the scanner room and placed into the MRI scanner. Right before the task started, participants were asked to repeat the task instructions, including the different cue-outcome associations for hit and miss trials, followed by another practice of the task within the scanner. The practice session was aborted when the participant had played at least one block of each condition. Then participants were told the following:

*“OK. Let’s start the real games. There will be two games, each about 7 minutes long. It is important to know that the computer won’t let you win all the time. So don’t get discouraged if you once in a while make a mistake or if you do not respond fast enough. Just move on and try to press the response button correctly the next time. Please use your right/left thumb (blue response button) to respond.”*

**Table S1**

List of favorite interests and hobbies by group.

| **TDC** | **ASD** |
| --- | --- |
| “Minecraft” video game (3x)  “Call of Duty” video game (2x)  “Pokemon” video game  “Kingdom Hearts” action role play  “Phillies” baseball team  “Philadelphia Flyers” ice hockey team  Kevin Durant basketball player  “Degrassi” TV series  “Family Guy” comic series  “Charmed” comic series  “Harry Potter” movies  “Divergent” movies  “Thousand Foot Krutch” music band  “Beatles” music band  “My Generation” dolls  Air guns  Fishing  Soccer  Filmmaking | “Minecraft” video game (5x)  “Pokemon” video game (4x)  “Phillies” baseball team (4x)  “Angry Birds” video game (3x)  “Sonic Generations” video game  “League of Legends” video game  “Rome: Total War” video game  “Call of Duty: Black Ops 2” video game  “Animal Crossings” video game  “Phineas & Ferb” comic series  “Sponge Bob” comic series  “Madoka Magica” comic series  “Transformers” movies  “My Favorite Martian” movie  “Power Rangers” movie  “Pirates of the Caribbean” movies  Race car model “Caparro T1”  “Lalaloopsy” doll  “Monopoly” board game  “Mancala” board game  Monster trucks  Geographic maps  Reptiles  Computers  Elevators  Cheerleading |

**Table S2**

Bivariate correlation coefficients between caudate responses for IR>SR and clinical symptom severity in the ASD group.

|  | _r_Caudate  IR>SR | _l_Caudate  IR>SR | _bilat_Caudate  IR>SR |
| --- | --- | --- | --- |
| _r_Caudate IR>SR |  | 0.80*** | 0.87*** |
| _l_Caudate IR>SR | 0.80*** |  | 0.99*** |
| _bilat_Caudate IR>SR | 0.87*** | 0.99*** |  |
| ADOS-2 _severity score_ | -0.02 | 0.04 | 0.01 |
| SRS-2 SCI _T-score_ | **0.31*** | 0.23 | 0.28 |
| ADOS-2 RRB _total score_ | 0.28 | **0.43**** | **0.41*** |
| SRS-2 RRB _T-score_ | 0.22 | 0.14 | 0.18 |
| ADI-R RRB _total score_ | -0.17 | -0.07 | -0.09 |
| RBS-R _total score_ | 0.10 | 0.21 | 0.28 |
| IS _total score_ | 0.28 | 0.19 | 0.24 |

ASD = autism spectrum disorder; IR>SR = interest reward > social reward; r = right; l = left; ADOS-2 = Autism Diagnostic Observation Scale – 2^nd^ Edition; ADOS-2 RRB = Autism Diagnostic Observation Scale – 2^nd^ Edition, Restricted and Repetitive Behaviors domain; ADI-R RRB = Autism Diagnostic Interview – Revised, Restricted and Repetitive Behaviors domain; SRS-2 SCI = Social Responsiveness Scale – 2^nd^ Edition, social communication and interaction subscale; SRS-2 RRB = Social Responsiveness Scale – 2^nd^ Edition, restricted interests and repetitive behavior subscale; RBS-R = Repetitive Behavior Scale-Revised; IS = Interests Scale.

**Table S3**

Whole-brain and ROI thresholds used in prior fMRI studies (cited in this paper) that investigated reward system activation in ASD versus TDC.

| **Study author (year) in alphabetical order** | **Whole-brain analyses** | **ROI analyses** |
| --- | --- | --- |
| Cascio et al. (2013) | Uncorrected p<0.005 at the voxel level (with ≥10 contiguous voxels) | Functional ROIs (FWE-corrected at p≤0.05) |
| Delmonte et al. (2012) | Uncorrected p<0.001 at the voxel level (with ≥10 contiguous voxels) | Structural ROIs (FWE-corrected at p≤0.05) |
| Dichter et al. (2012a) | Uncorrected p<0.005 at the voxel level (with ≥10 contiguous voxels) | Not done/reported |
| Dichter et al. (2012b) | Uncorrected p<0.01 at the voxel level as the cluster-defining threshold, cluster-corrected at p<0.05 using Gaussian RFT | Not done/reported |
| Kohls et al. (2013) | Uncorrected p<0.01 at the voxel level as the cluster-defining threshold, cluster-corrected at p<0.05 using Gaussian RFT | Structural ROIs (FWE-corrected at p≤0.05) |
| Scott-Van Zeeland et al. (2012) | Uncorrected p<0.01 at the voxel level as the cluster-defining threshold, cluster-corrected at p<0.05 using Gaussian RFT | Functional ROI (uncorrected p<0.005 at the voxel level with ≥20 contiguous voxels) |
| *Present study* | *Uncorrected p<0.001 at the voxel level as the cluster-defining threshold, cluster-corrected at p<0.05 using Gaussian RFT* | *Structural ROIs (FWE-corrected at p≤0.05)* |

ASD = autism spectrum disorder; TDC = typically developing controls; ROI = region of interest; FWE = family-wise error rate; RFT = random field theory.
